# Pseudoanginal chest pain associated with vagal nerve stimulation: a case report

**DOI:** 10.1186/s12883-020-01693-5

**Published:** 2020-04-20

**Authors:** James B. Nichols, Abigail P. McCallum, Nicolas K. Khattar, George Z. Wei, Rakesh Gopinathannair, Haring J. W. Nauta, Joseph S. Neimat

**Affiliations:** 1 Department of Neurological Surgery, University of Louisville School of Medicine, Louisville, KY USA; 2 Kansas Heart Rhythm Institute, HCA Midwest Health, Overland Park, KS USA

**Keywords:** Vagal nerve stimulation, Typical angina, Spinothalamic tract, Convergence, Epilepsy

## Abstract

**Background:**

Vagal nerve stimulation (VNS) can be an effective therapy for patients with epilepsy refractory to anti-epileptic drugs or intracranial surgery. While generally well tolerated, it has been associated with laryngospasm, hoarseness, coughing, dyspnea, throat and atypical chest pain, cardiac symptoms such as bradycardia and occasionally asystole. We report on a patient receiving vagal nerve stimulation who experienced severe typical anginal chest pain during VNS firing without any evidence of cardiac ischemia or dysfunction. Thus, the pain appeared to be neuropathic from the stimulation itself rather than nociceptive secondary to an effect on heart function.

**Case presentation:**

A 29-year-old man, with a history of intractable frontal lobe epilepsy refractory to seven anti-epileptic medications and subsequent intracranial surgery, underwent VNS implantation without complications. On beginning stimulation, he began to have intermittent chest pain that corresponded temporally to his intermittent VNS firing. The description of his pain was pathognomonic of ischemic cardiac chest pain. On initial evaluation, he displayed Levine’s sign and reported crushing substernal chest pain radiating to the left arm, as well as shortness of breath walking upstairs that improved with rest. He underwent an extensive cardiac workup, including 12-lead ECG, cardiac stress test, echocardiogram, 12-day ambulatory cardiac monitoring, and continuous ECG monitoring each with and without stimulation of his device. The workup was consistently negative. Inability to resolve the pain necessitated the disabling and eventual removal of the device.

**Conclusion:**

To our knowledge, this is the first report of pseudoanginal chest pain associated with VNS. This occurrence prompted our review of the mechanisms of cardiac chest pain and suggests that vagal afferents may convey anginal pain separately or in parallel with known spinal cord pain mechanisms. These insights into the physiology of chest pain may be of general interest and important to surgeons implanting VNS devices who may potentially encounter such symptoms.

## Background

Vagal nerve stimulation (VNS) can be effective for patients with epilepsy refractory to anti-epileptic drugs or intracranial surgery, or those who are poor surgical candidates [1]. It has been used to treat refractory epilepsy since the early 1990s, and has recently shown some benefit in cases of refractory depression and Alzheimer’s. Multiple randomized trials have shown VNS therapy to be efficacious in treating refractory epilepsy, with 50–60% of patients experiencing a 50% or greater reduction in seizure activity [2, 3]. While some adverse effects have been reported; including hoarseness, cough, throat pain, or dyspnea, VNS therapy is generally well tolerated. The more serious side effects that have been reported include bradycardia and asystole. For these symptoms, an intraoperative screening is routinely performed and, if absent, such cardiac effects have rarely recurred [4–8].

Antiseizure activity of VNS therapy is thought to be due to stimulation of A- and B- nerve fibers contained within the vagus nerve that travel to the solitary nucleus [9]. However, stimulation can cause a variety of side effects due to the diverse functions of the vagus nerve. The reported cardiac side effects of VNS (heart block, and asystole) have been attributed to stimulation of descending vagal parasympathetic fibers to the heart affecting conduction at the SA-node. Mechanisms of cardiac nociception are significantly more complicated and have not traditionally been modulated with vagus nerve stimulation. The occurrence of such pseudoanginal pain in this patient prompted our review of the pertinent anatomy and physiology.

## Case presentation

A 29-year-old man with a history of intractable frontal lobe epilepsy was treated unsuccessfully with a variety of anti-epileptic drugs (AED), including valproic acid, oxcarbazepine, levetiracetam, clobazam, lacosamide, lamotrigine and zonisamide. Upon presentation, the patient was on brivaracetam 100 mg twice daily, topiramate 350 mg daily, and eslicarbazepine 800 mg daily. Because AEDs failed to control his seizures, he underwent subdural grid monitoring and resection of the inferior frontal gyrus and orbital frontal cortex. He was seizure-free for approximately 6 months but experienced seizure recurrence as frequently as seven episodes per month. The semiology of the seizures was consistent with his pre-resection epilepsy. Electroencephalography (EEG) demonstrated broad minor interictal discharges but a clear additional seizure focus was not obvious. The patient was offered VNS therapy for symptomatic relief. The patient underwent VNS placement without intra- or perioperative complications. The helical coil was placed in the correct orientation, the patient experienced typical laryngospasm and the impedance was within normal limits. The initial device settings can be seen in Table 1.

**Table 1:** Initial VNS device setings

Parameter	Setting
Output Current	0.25 mA
Signal Frequency	30 Hz
Pulse Width	500
On Time	30 s
Off Time	5 min
Mag Current	0.5 mA
Mag On Time	60
Mag Pulse Width	500
Autostim Current	0.375
On Time	60
Pulse Width	500
Tachycardia	On
Threshold	40%

One week after stimulation was initiated, the patient began experiencing intermittent crushing substernal chest pain localized to the left lateral sternal border which radiated to the left arm, with associated numbness. The patient reported shortness of breath on exertion and also exhibited Levine’s sign (describing the pain with his fist clenched in front of his chest) on clinical evaluation. Symptoms only occurred upon VNS activation, resolved shortly after VNS deactivation, and did not occur when the device was turned off. His pain, however, was not constant or time locked to periods of stimulation, suggesting that stimulation alone was not sufficient to cause his pain or that it did so variably. Because the patient described angina pain so clearly with further suggestion of cardiac modulation manifest as dyspnea on exertion, the VNS was, therefore, inactivated pending further cardiac workup.

**Fig. 1:**
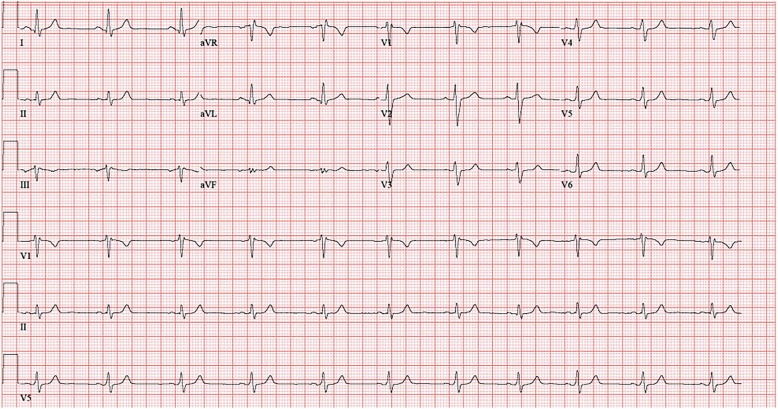
Baseline ECG of the patient that showed a benign incomplete right bundle branch block

The patient reported that the pseudo-anginal pain was distinct from the typical laryngospasm associated with vagal stimulation which he also experienced but was tolerable. He had no known cardiac history or significant risk factors and was otherwise in excellent health. Given the severity of his symptoms we conducted a full cardiac workup. This included a 12-lead ECG, treadmill stress test echocardiography, and ambulatory ECG monitoring. The workup was negative except for an incomplete right bundle branch block found on ECG and present with or without VNS stimulation. This finding was not considered clinically relevant to the reported symptoms. Baseline ECG tracing can be seen in Fig. 1.

He subsequently underwent monitored re-activation of his VNS with continuous ECG monitoring in the cardiac laboratory. Stimulation reproduced the substernal chest pain. No new ECG abnormalities were observed either on 12 lead ECG or during an extended halter monitor evaluation. Due to the debilitating nature of the chest pain, the VNS system was removed per the patient’s request.

## Discussion and conclusions

Herein, we report a case of VNS-induced pseudoanginal chest pain. To our knowledge, this has not been previously reported.

Multiple case reports have described intraoperative and postoperative bradycardia, asystole, and arrhythmias associated with VNS placement and activation [7, 8, 10]. These symptoms have been attributed to stimulation of descending parasympathetic fibers in the left vagus nerve that innervate the sinoatrial (SA) node. We review putative mechanisms underlying typical anginal pain in order to understand the likely mechanisms involved in its generation.

Cardiac anginal pain is a complex phenomenon probably mediated through several sensory pathways. Foreman et al. describe anginal pain as primarily conveyed through sympathetic spinal afferents with cell bodies located in dorsal root ganglion. The primary axons travel in the tract of Lissauer to synapse on secondary neurons which then issue axons ascending within the spinothalamic tract (STT) to the thalamus. The somatic symptoms experienced during anginal pain are attributed to somatic and visceral afferent fibers that converge on a common pool of STT cells resulting in referred pain to somatic structures, manifest as sternal or radiating left arm pain [11].

Spinal cord stimulation (SCS) strategies have been developed to target the STT neurons to reduce their activity and alleviate angina caused by coronary ischemia [12]. More specifically, there is evidence to show that SCS directly inhibits cardiac nociception, but also indirectly improves cardiac function by blood flow redistribution and reduction of cardiomyocyte oxygen demand, which are thought to be the primary factors for triggering ischemic angina [12–14].

**Fig. 2:**
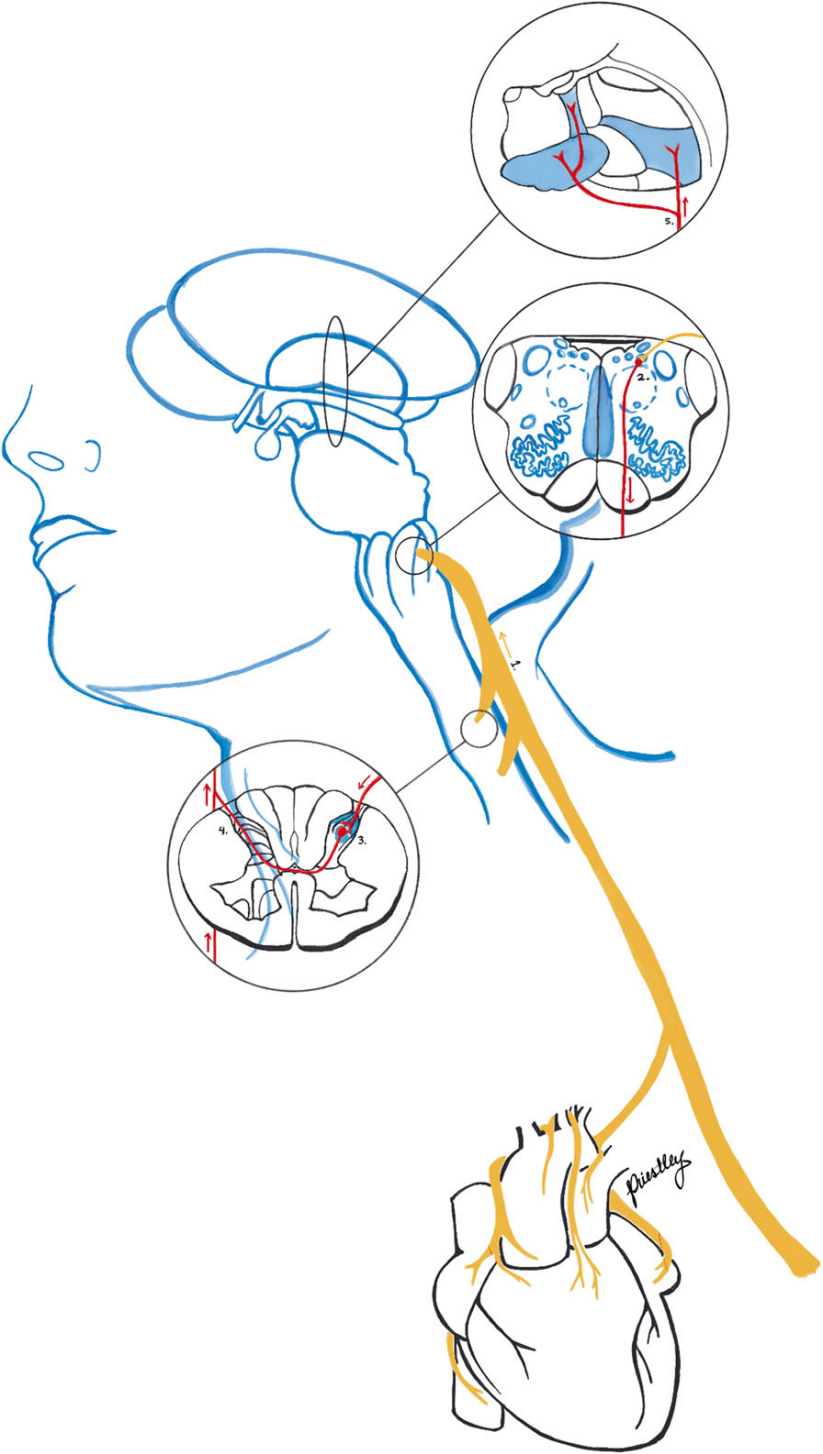
Schematic drawing of the vagal pain pathway. 1: Vagus nerve 2: Nucleus of the solitary tract in the medulla oblongata 3: Descending parasympathetic fibers at the level of C1–2 entering the dorsal horn of the spinal cord 4: Spinothalamic neurons exiting the spinal cord en route to the thalamus 5: Projections of the spinothalamic neurons to the centralis lateralis, centrum medianum parafascicularis and ventral posterolateral nuclei of the thalamus

Foreman et al. also describe a secondary nociceptive pathway transmitted through vagal afferents. Such fibers innervating the heart, synapse in the nucleus of the solitary tract (NTS). From there, secondary neurons descend to C1–2 to terminate on STT neurons (Fig. 2) [11]. This mechanism is similar to that implicated in glossopharyngeal neuralgia [11]. The post-synaptic dorsal column (PSDC) pathway is involved in cardiac mechanoreception but, there is no evidence that it is involved in cardiac pain [15].

Sensory endings of primarily unmyelinated vagal afferents are distributed throughout cardiac regions. Incidentally, a greater concentration of vagal nerve endings has been found to innervate the infero-posterior wall of the left ventricle. Ischemia and infarction to this region of the heart lead to an increased vagal response resulting in bradycardia, hypotension and nociception [11].

Middlekauf et al. described the action of adenosine on A1 receptors of cardiac vagal afferents to mediate nociception during ischemic events [9, 16]. Evidence of integration of nociceptive inputs occurs at the level of the neurons of the STT. However, additional integration centers have been involved including the parabrachial nucleus, peri-locus coeruleus nucleus as well as the raphe magnus nucleus [11]. Visceral and somatic fibers course to the thalamus via the STT, specifically to the ventroposterolateral (VPL) nucleus in the lateral thalamus and the centralis lateralis (CL) and centrum medianum-parafascicularis nuclei (CM) in the intralaminar thalamus [9]. Nociceptive information is combined into common central neurons to higher regions of the cortex and amygdala [11]. Information relayed to the medial and lateral thalamus is communicated to the somatosensory cortex where the specifics of painful stimuli, such as location, duration, and intensity can be processed. Pathways to the amygdala, insula and cingulate gyrus play a role in the emotional perception of painful stimuli and can intensify the nociceptive perception [11]. It is important to note that vagal nerve stimulation in our patient has selectively activated the afferent cardiac visceral nociceptive component of the vagus nerve without any other changes, whether retro- or anterograde.

In summary, typical cardiac anginal pain is experienced by the activation of various nociceptive pathways. However, vagal nerve stimulation does not explain the activation of all the aforementioned pathways involved in the genesis of true anginal pain. Further research is needed to characterize the convergent connections involved in the mediation of cardiac pain.

Despite VNS being well-tolerated in most patients, it is important to be aware that it may trigger pseudoanginal symptoms in the absence of actual insults to the myocardium. As with any cardiac symptoms, an extensive work up to rule out an ischemic cause is certainly warranted (as was done in this patient) before dismissing such symptoms as “pseudoanginal.” In rare patients such pain may limit the ability to continue VNS therapy for seizure reduction.

## Data Availability

The datasets used and/or analyzed during the current study are available from the corresponding author on reasonable request.

## Abbreviations

VNS: Vagal nerve stimulator
ECG: Electrocardiogram
AED: Anti-epileptic drugs
SA: Sino-atrial node
STT: Spinothalamic tract
PSDC: Post-synaptic dorsal column
SCS: Spinal cord stimulation
VPL: Ventro-posterolateral nucleus
CM: Centrum medianum-parafascicularis nucleus
